# The potency of psychiatric questionnaires to distinguish major mental disorders in Chinese outpatients

**DOI:** 10.3389/fpsyt.2022.1091798

**Published:** 2022-12-22

**Authors:** Jiayi Wang, Enzhao Zhu, Pu Ai, Jun Liu, Zhihao Chen, Feng Wang, Fazhan Chen, Zisheng Ai

**Affiliations:** ^1^School of Medicine, Tongji University, Shanghai, China; ^2^School of Business, East China University of Science and Technology, Shanghai, China; ^3^Clinical Research Center for Mental Disorders, Shanghai Pudong New Area Mental Health Center, School of Medicine, Chinese-German Institute of Mental Health, Tongji University, Shanghai, China; ^4^Department of Medical Statistics, School of Medicine, Tongji University, Shanghai, China

**Keywords:** psychiatric questionnaires, mental disorders, machine learning (ML), Symptom Checklist-90 (SCL-90), Hamilton Anxiety Rating Scale (HAM-A), Hamilton Depression Rating Scale (HAM-D)

## Abstract

**Background:**

Considering the huge population in China, the available mental health resources are inadequate. Thus, our study aimed to evaluate whether mental questionnaires, serving as auxiliary diagnostic tools, have efficient diagnostic ability in outpatient psychiatric services.

**Methods:**

We conducted a retrospective study of Chinese psychiatric outpatients. Altogether 1,182, 5,069, and 4,958 records of Symptom Checklist-90 (SCL-90), Hamilton Anxiety Rating Scale (HAM-A), and Hamilton Depression Rating Scale (HAM-D), respectively, were collected from March 2021 to July 2022. The Mann–Whitney U test was applied to subscale scores and total scores of SCL-90, HAM-A, and HAM-D between the two sexes (male and female groups), different age groups, and four diagnostic groups (anxiety disorder, depressive disorder, bipolar disorder, and schizophrenia). Kendall's tau coefficient analysis and machine learning were also conducted in the diagnostic groups.

**Results:**

We found significant differences in most subscale scores for both age and gender groups. Using the Mann–Whitney U test and Kendall's tau coefficient analysis, we found that there were no statistically significant differences in diseases in total scale scores and nearly all subscale scores. The results of machine learning (ML) showed that for HAM-A, anxiety had a small degree of differentiation with an AUC of 0.56, while other diseases had an AUC close to 0.50. As for HAM-D, bipolar disorder was slightly distinguishable with an AUC of 0.60, while the AUC of other diseases was lower than 0.50. In SCL-90, all diseases had a similar AUC; among them, bipolar disorder had the lowest score, schizophrenia had the highest score, while anxiety and depression both had an AUC of approximately 0.56.

**Conclusion:**

This study is the first to conduct wide and comprehensive analyses on the use of these three scales in Chinese outpatient clinics with both traditional statistical approaches and novel machine learning methods. Our results indicated that the univariate subscale scores did not have statistical significance among our four diagnostic groups, which highlights the limit of their practical use by doctors in identifying different mental diseases in Chinese outpatient psychiatric services.

## 1. Introduction

Approximately 15% of the world's working population is estimated to experience a mental disorder at any given time. Mental disorders are a leading cause of disease burden worldwide, and can have a substantial financial impact on patients and their households. In disability-adjusted life-years, depressive and anxiety disorders ranked 13th and 24th among the leading causes of disease burden worldwide (1). According to the Global Burden of Diseases (GBD), Injuries, and Risk Factors Study, an acute state of schizophrenia has the highest disability weight (2). All the above-mentioned diseases and bipolar disorder (3) not only injure patients' health but also raise the risk of suicide or other adverse health outcomes.

In 2019, 970 million people lived with mental diseases worldwide, among which anxiety disorders, depressive disorders, schizophrenia, and bipolar disorders occurred in 301, 280, 24, and 40 million people, respectively. The situation worsened after the COVID-19 pandemic; the estimated number of people with major depressive disorder has increased from 193 million to 246 million, and the number of people with anxiety disorders has increased to 374 million (4).

In China, the economy has developed rapidly and tremendous social change has happened in recent decades, which is likely to have resulted in a considerable increase in the prevalence of general mental diseases. In 2016, the prevalence of any mental disease was 16.6% during lifetime, and the 1-year prevalence was 9.3% (5). As a result, the demand for hospitalization and psychiatric outpatient services has grown every year.

Considering the huge population and high prevalence of mental disorders in China, mental health resources and labor force are both insufficient. According to a study of 41 top psychiatric hospitals in 29 provinces, the overall ratio of psychiatrists was 0.16 per bed, and only 31.7% of these hospitals attained the lower limit of the governing psychiatric staff per bed ratio. In addition, meeting patients' requirements in outpatient clinics has been a general problem for decades because of the unbalanced mental health resources and labor force in China. Although the study showed that each psychiatrist saw 7–45 patients on average every working day, the actual workload was much heavier, considering that they also included psychiatrists who did not work in outpatient services in the total number (6). Chinese psychiatrists had a heavy workload as they diagnosed and cared for too many patients per day, and the COVID-19 pandemic even exacerbated this imbalance between supply and demand of medical resources (7). Therefore, there is an urgent need to increase the application of efficient tools.

Mental questionnaires have been widely used since the last century, and many have shown great reliability and validity. However, to date, the overall assessment of mental questionnaires used in outpatient psychiatric clinics has been insufficient, which highlighted the importance of further research.

In recent years, several studies have focused on the application of artificial intelligence (AI). A 2022 study used a machine learning (ML) model to assess the differential item functioning of KINDL among children with and without attention-deficit/hyperactivity disorder (8). Wang et al. obtained users' comments from social media and used a language model to discriminate whether they might have depression (9). Basaia et al. built an ML model using 3D T1-weighted magnetic resonance imaging (MRI) to detect whether a subject is healthy, has mild cognitive impairment, or is diagnosed with Alzheimer's disease (10).

Thus, in this study, we used ML as an enlightening and testing tool rather than a predictive tool, inspired by a recent study (11). The study suggested that a simple ML model can help identify the potential patterns between mathematical objects, which corresponds with our aim to discover the latent features extracted from the subscale scores of mental questionnaires, which may be useful to differentiate mental diseases.

In conclusion, this study aimed to evaluate whether these questionnaires have significant diagnostic efficacy in an actual hospital environment and to relieve the burden of psychiatrists and help more people get diagnosed and treated. We are the first to conduct wide and comprehensive analyses on the use of mental questionnaires in Chinese outpatient clinics with the combination of traditional statistical approaches and novel ML models.

## 2. Methods

### 2.1. Study design

We conducted a retrospective study on the diagnostic effects of commonly used mental health questionnaires in psychiatric outpatients. The Symptom Checklist-90 (SCL-90), Hamilton Anxiety Rating Scale (HAM-A), and Hamilton Depression Rating Scale (HAM-D) questionnaire records were extracted from the database of the Pudong Mental Health Center, Shanghai, China, which can provide a large and representative sample of Chinese psychiatric outpatients. The duration was from March 2021 to July 2022. Altogether, 11,209 records comprising 1,182, 5,069, and 4,958 records of SCL-90, HAM-A, and HAM-D, respectively, were collected.

**Figure 1:**
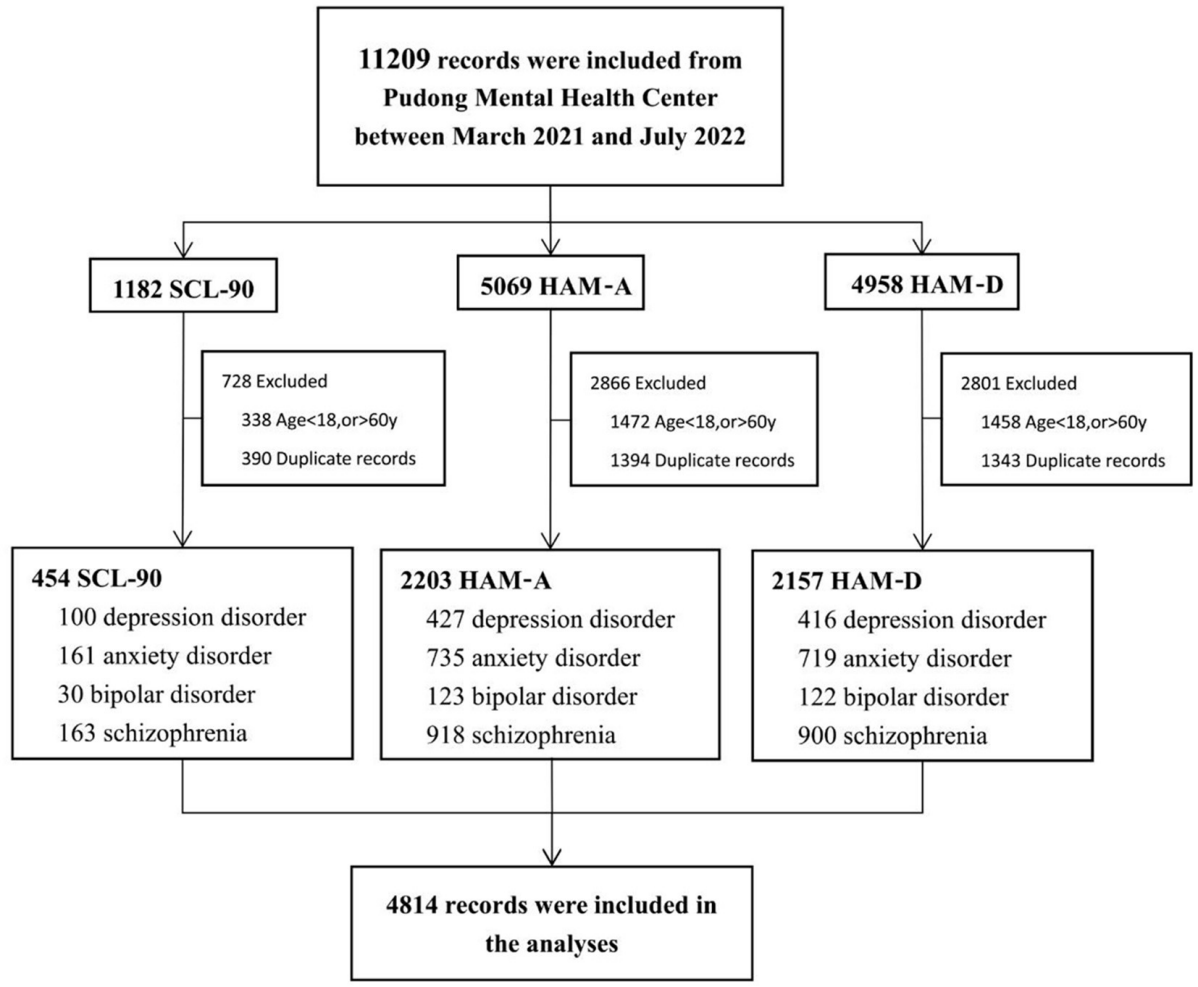
Flow chart of experimental design. HAM-A, Hamilton Anxiety Rating Scale; HAM-D, Hamilton Depression Rating Scale; SCL-90, Symptom Checklist-90.

The diagnoses of these patients were classified according to the Diagnostic and Statistical Manual of Mental Disorders, Fifth Edition (DSM-5). The top four diseases, by number, were depressive disorder, anxiety disorder, bipolar disorder, and schizophrenia. Records of patients aged 18–60 years with the above four diseases were included, while the others were excluded. Thus, 943, 1,615, 275, and 1,981 records with diagnoses of anxiety disorder, depressive disorder, bipolar disorder, and schizophrenia and 454, 2,203, and 2,157 records of SCL-90, HAM-A, and HAM-D, respectively, were included, which was a total of 4,814 (42.95%) records (male: 1,573, 32.68%; female: 3,241, 67.32%) (Table 1, Figure 1).

**Table 1:** Demographic characteristics.

**Characteristic[Missing]**	**SCL-90**	**HAM-A**	**HAM-D**	**Total**
Number of records	454	2,203	2,157	4,814
Age, median (IQR), y	36.0 (25.0–50.0)	41.0 (29.0–57.0)	41.0 (29.0–57.0)	
Education level [190]				4,624
High school or lower	188 (43.5%)	964 (46.0%)	974 (46.5%)	2,126
College or higher	244 (56.5%)	1,133 (54.0%)	1,121 (53.5%)	2,498
**Sex, No. (%)**				
Male	159 (35.0%)	720 (32.7%)	694 (32.2%)	1,573
Female	295 (65.0%)	1,483 (67.3%)	1,463 (67.8%)	3,241

SCL-90, Symptom Checklist-90; HAM-A, Hamilton Anxiety Rating Scale; HAM-D, Hamilton Depression Rating Scale.

Data (age) are presented as median and interquartile range (IQR).

The study was approved by the ethics committee of the Institutional Review Board at Pudong Mental Health Center.

### 2.2. Questionnaires

#### 2.2.1. Symptom Checklist-90

The SCL-90 is a self-rating psychiatric symptom scale based on the Hopkins Symptom List (HSCL1973) compiled by Derogatis (12). It has the advantage of being simple and easy to measure. The scale consists of 90 items and each scores on a five-point scale, representing the severity of symptoms (0 = never, 1 = mild, 2 = moderate, 3 = severe, and 4 = severe), assessing nine dimensions: somatization (SOM, 12 items), obsessive-compulsion (OC, 10 items), interpersonal sensitivity (IS, 9 items), depression (DEP, 13 items), anxiety (ANX, 10 items), hostility (HOS, 6 items), phobic anxiety (PHOB, 7 items), paranoia (PAR, 6 items), and psychosis (PSY, 10 items). The additional items (ADD, 7 items) were mainly used to reflect the status of sleep and diet (13, 14). The mean score of the questionnaire is a global index of distress called the Global Severity Index (GSI). The Chinese version of SCL-90 has good reliability and validity (12).

#### 2.2.2. Hamilton Anxiety Rating Scale

The HAM-A is the most frequently used clinician-administered scale for measuring overall anxiety. It consists of 14 items, which can be divided into two subscales: “somatic anxiety” and “psychic anxiety.” Each scale item was scored from 0 to 4, with the total score ranging from 0 to 70. Higher scores indicate higher levels of anxiety. The Chinese version of HAM-A has good reliability and validity (15).

#### 2.2.3. Hamilton Depression Rating Scale

The HAM-D is a 17 items checklist to evaluate symptoms in the past 7 days. It is the most commonly used clinician-related scale to assess depression severity in people with depressive disorder and has remained the gold standard for decades (16). Each item is ranked 0–4 or 0–2, and total score range is 0–61. Scores < 7 and >24 represent severe depression and the absence of depression, respectively. These 17 items can be divided into 7 subscales: “anxiety,” “weight,” “cognitive disorder,” “daily change,” “obstruction,” “sleep disorder,” and “despair.” Higher scores indicate higher levels of depression (17). The Chinese version of HAM-D has good reliability and validity (15).

### 2.3. Statistics

All statistical analyses were performed using R 4.1.3. Continuous variables are expressed as median and interquartile range (IQR), while categorical variables are expressed as number and composition ratio (%). Continuous variables were compared using the Welch's *t*-test (if normal) and Mann–Whitney U test (if non-normal). The constituent ratios and rates were compared using the chi-square test and Fisher's exact test, as appropriate. As our data distribution was not normal, we conducted Kendall's tau partial correlation analyses to test for correlations between item scores and diseases, which can eliminate the effects of age and gender. Statistical significance was set at *P* < 0.05.

### 2.4. Machine learning

We used four ML models and trained them on each scale separately. The Self-Attention and Intersample Attention Transformer (SAINT) (18) is a state-of-the-art DL model specialized for structured data. SAINT projects all the inputs into a combined vector space in which self-attention is conducted. The difference between SAINT and other transformer models (19) is that SAINT concatenates the embeddings of each training feature of each sample, and then computes attention over samples, which is called intersample attention. A fully connected neural network (FCNN) is a common DL model with several plain fully connected layers. XGBoost (20) is a type of gradient boosted decision tree (GBDT) that is frequently used in classification and regression tasks. Logistic regression (LR) is a common model that is used to calculate odds ratios and is competent in classification tasks.

We allocated 75% of the samples as the training set and 25% of the samples as the testing set, using a stratified random sampling strategy to ensure that the proportion of diseases in the testing set were identical to those in the training set. We utilized 3-folds-cross-validation to train and validate the models in the training set, which meant models would back-propagate the gradient in two-thirds of the training set, while validating in the remaining one-third of the training set. The testing set was unseen by the models during the training period and was used to evaluate the predictive value of the models. We used grid search in the training set to tune the hyperparameters of the models. As for DL models, we recorded the loss of the validation set during each round of iterations, if it did not decrease in 1,000 iterations, the training process would be automatically terminated.

## 3. Results

### 3.1. Demographic statistics

Altogether, 4,814 records were collected in this study, among which, 3,241 were female (67.3%) and 1,573 were male (32.7%); the majority of them had an education level of college or higher (2,498, 54.0%). Four hundred fifty-four records of SCL-90 were included, of which 159 (35.0%) were male, 295 (65.0%) were female, and the quartile age was 36.0 (25.0–50.0) years; 2,203 records of HAM-A were included comprising 720 males (32.7%) and 1,483 females (67.3%), with a quartile age of 41.0 (29.0–57.0) years; and 2,157 records of HAM-D were included of which 694 were male (32.2%), 1,463 were female (67.8%), and the quartile age was 41.0 (29.0–57.0) years. The number of records with diagnoses of anxiety disorder, depressive disorder, bipolar disorder, and schizophrenia were 100, 161, 30, and 163 in the SCL-90 dataset, 427, 735, 123, and 918 in the HAM-A dataset, and 416, 719, 122, and 900 in the HAM-D dataset, respectively. The detailed demographic information is presented in Table 1.

### 3.2. Questionnaires' results

#### 3.2.1. Analyses of demographic factors and scale scores

##### 3.2.1.1. Gender group

In the datasets of three questionnaires, participants were grouped by gender as “male” or “female.” The Mann–Whitney U test was conducted for subscale scores and the total scores of SCL-90, HAM-A, and HAM-D between the two gender groups.

In HAM-A, the total score [male = 14, (9–19), female = 14, (11–21), *P* < 0.001], somatic anxiety [male = 6, (3–8), female = 6, (3–9), *P* < 0.001], and psychic anxiety [male = 8, (6–11), female = 8, (6–12), *P* < 0.001] scores all showed statistical differences. In HAM-D, the total score [male = 19, (12–26), female = 20, (14–27.5), *P* = 0.0044], anxiety [male = 5, (3–7), female = 6, (4–7), *P* = 0.0013], obstruction [male = 4, (2–5), female = 4, (2–5), *P* = 0.0004], and sleep disorder [male = 3 (2–5), female = 4, (2–6), *P* = 0.0303] scores all showed statistical differences. Weight, cognitive disorder, daily changes, and despair scores did not show significant differences. In SCL-90, except for somatization score [male = 1.5, (1.17–2.25), female = 1.75, (1.25–2.58), *P* = 0.0444], we did not find significant differences between the two groups in the global severity index and other subscale scores.

In addition, the female group showed higher scores on many subscales. In HAM-A, females had a higher upper quartile than males in somatic anxiety score (9.0 vs. 8.0, *P* < 0.001) and psychic anxiety scores (12 vs. 11, *P* < 0.001), and also had a higher lower quartile (11 vs. 9, *P* < 0.001) and upper quartile (21 vs. 19, *P* < 0.001) of the total score. In HAM-D, females had higher scores than males in the total score [20, (14–27.5) vs. 19, (12–26), *P* = 0.0044], anxiety score [6, (4–7) vs. 5, (3–7), *P* = 0.0013], and sleep disorder score [4, (2–6) vs. 3, (2–5), *P* = 0.0303]. In SCL-90, females had higher somatization scores than males [1.75, (1.25–2.58) vs. 1.5, (1.17–2.25), *P* = 0.0044] (Table 2).

**Table 2:** Mann–Whitney U test for gender groups.

	**Male**	**Female**	***P*-value**
**HAM-A**			
Number of records (*n*, %)	720	1,483	
Total	14.0 (9.0–19.0)	14.0 (11.0–21.0)	< 0.0001^***^
Somatic anxiety	6.0 (3.0–8.0)	6.0 (3.0–9.0)	< 0.0001^***^
Psychic anxiety	8.0 (6.0–11.0)	8.0 (6.0–12.0)	0.0003^***^
**HAM-D**			
Number of records (*n*, %)	694	1,463	
Total	19.0 (12.0–26.0)	20.0 (14.0–27.5)	0.0044^**^
Anxiety	5.0 (3.0–7.0)	6.0 (4.0–7.0)	0.0013^**^
Weight	0 (0–1.0)	0 (0–1.0)	0.4972
Cognitive disorder	3.0 (1.0–5.0)	3.0 (2.0–5.0)	0.1169
Daily change	0 (0–1.0)	0 (0–1.0)	0.4387
Obstruction	4.0 (2.0–5.0)	4.0 (2.0–5.0)	0.0004
Sleep disorder	3.0 (2.0–5.0)	4.0 (2.0–6.0)	0.0303^*^
Despair	3.0 (1.0–4.0)	3.0 (1.0–4.0)	0.0734
**SCL-90**			
Number of records (*n*, %)	159	295	
Global severity index	2.0 (1.5–2.7)	2.1 (1.5–2.9)	0.4677
Somatization	1.5 (1.2–2.3)	1.8 (1.3–2.6)	0.0444^*^
Compulsive	2.2 (1.5–3.1)	2.3 (1.5–3.2)	0.8589
Interpersonal sensitivity	2.0 (1.4–2.8)	2.0 (1.4–3.2)	0.3672
Depression	2.3 (1.5–3.3)	2.5 (1.6–3.6)	0.1577
Anxiety	2.2 (1.4–3.2)	2.3 (1.5–3.2)	0.3518
Hostility	2.0 (1.3–2.8)	1.8 (1.3–3.0)	0.9407
Phobic anxiety	1.6 (1.1–2.4)	1.6 (1.1–2.4)	0.5140
Paranoid ideation	1.8 (1.2–2.8)	1.8 (1.2–2.7)	0.9541
Psychoticism	1.8 (1.3–2.5)	1.8 (1.2–2.7)	0.9829
Additional item-sleep and diet	2.3 (1.6–3.0)	2.1 (1.6–3.1)	0.9413

HAM-A, Hamilton Anxiety Rating Scale; HAM-D, Hamilton Depression Rating Scale; SCL-90, Symptom Checklist-90.

Data are presented as median and interquartile range (IQR).

Statistical analysis: Mann–Whitney U test; ^*^P < 0.05, ^**^P < 0.005, ^***^P < 0.0001.

##### 3.2.1.2. Age group

Then, participants were grouped by ages as group A1 (early adulthood, 18–35 y), group B1 (adulthood, 35–50 y), and group C1 (middle age, 50–65 y), and the Mann–Whitney U test was applied. Significant differences between the three age groups were found in nearly all the total scale and subscale scores, except for total and physical anxiety scores in HAM-A, and total and weight scores in HAM-D.

Among the age groups, the somatic anxiety [7, (4–9)] score of HAM-A and anxiety score [6, (4–8)] of HAM-D were highest in the middle-age group. The early adulthood group had the highest cognitive disorder [4, (2–6)], daily change [1, (0–1)] scores in HAM-D, and highest GSI [2.56, (1.84–3.27)], SOM [1.92, (1.33–2.75)], OC [2.80, (2.00–3.60)], IS [2.67, (1.89–3.67)], DEP [3.00, (2.00–3.85)], ANX [2.70, (1.90–3.50)], HOS [2.33, (1.50–3.33)], PHOB [2.00, (1.29–2.86)], PAR [2.33, (1.67–3.17)], PSY [2.20, (1.60–3.10)], and ADD [2.60, (1.90–3.30)] scores in SCL-90 (Table 3).

**Table 3:** Mann–Whitney U test for age groups.

	**Early adulthood**	**Adulthood**	**Middle age**	***P*-value**
**HAM-A**				
Number of records (*n*, %)	802	608	793	
Total	14.0 (9.0–20.0)	14.0 (9.0–20.0)	14.0 (11.0–21.0)	0.0553
Somatic anxiety	5.5 (2.0–8.0)	6.0 (3.0–8.0)	7.0 (4.0–9.0)	< 0.0001^**^
Psychic anxiety	8.0 (6.0–12.0)	8.0 (6.0–12.0)	8.0 (6.0–12.0)	0.0904
**HAM-D**				
Number of records (*n*, %)	758	597	802	
Total	20.0 (13.0–28.0)	19.0 (13.0–26.0)	21.0 (13.0–27.0)	0.3388
Anxiety	5.0 (3.0–7.0)	5.0 (3.0–7.0)	6.0 (4.0–8.0)	0.0006^*^
Weight	0 (0–1.0)	0 (0–1.0)	0 (0–1.0)	0.6590
Cognitive disorder	4.0 (2.0–6.0)	3.0 (1.0–5.0)	3.0 (1.0–5.0)	< 0.0001^**^
Daily change	1.0 (0–1.0)	0 (0–1.0)	0 (0–1.0)	< 0.0001^**^
Obstruction	4.0 (2.0–5.0)	4.0 (2.0–5.0)	4.0 (2.0–5.0)	0.0002^*^
Sleep disorder	3.0 (1.0–5.0)	4.0 (2.0–5.0)	4.0 (3.0–6.0)	< 0.0001^**^
Despair	3.0 (1.0–4.0)	3.0 (1.0–4.0)	3.0 (1.0–4.0)	0.0032^*^
**SCL-90**				
Number of records (*n*, %)	217	120	117	
Global severity index	2.6 (1.8–3.3)	2.0 (1.5–2.8)	1.5 (1.2–1.9)	< 0.0001^**^
Somatization	1.9 (1.3–2.8)	1.7 (1.2–2.6)	1.5 (1.2–2.2)	0.0062^*^
Compulsive	2.8 (2.0–3.6)	2.3 (1.5–3.0)	1.5 (1.3–2.0)	< 0.0001^**^
Interpersonal sensitivity	2.7 (1.9–3.7)	2.0 (1.4–2.9)	1.4 (1.1–1.8)	< 0.0001^**^
Depression	3.0 (2.0–3.9)	2.4(1.5–3.5)	1.8 (1.3–2.5)	< 0.0001^**^
Anxiety	2.7 (1.9–3.5)	2.3 (1.4–3.2)	1.7 (1.3–2.2)	< 0.0001^**^
Hostility	2.3 (1.5–3.3)	1.8 (1.3–2.8)	1.3 (1.2–1.8)	< 0.0001^**^
Phobic anxiety	2.0 (1.3–2.9)	1.4 (1.1–2.3)	1.1 (1.0–1.6)	< 0.0001^**^
Paranoid ideation	2.3 (1.7–3.2)	1.7 (1.2–2.5)	1.2 (1.0–1.8)	< 0.0001^**^
Psychoticism	2.2 (1.6–3.1)	1.8 (1.3–2.5)	1.2 (1.0–1.7)	< 0.0001^**^
Additional item-sleep and diet	2.6 (1.9–3.3)	2.1 (1.5–3.0)	2.0 (1.3–2.6)	< 0.0001^**^

HAM-A, Hamilton Anxiety Rating Scale; HAM-D, Hamilton Depression Rating Scale; SCL-90, Symptom Checklist-90.

Data are presented as median and interquartile range (IQR).

Statistical analysis: Mann–Whitney U test; ^*^P < 0.005, ^**^P < 0.0001.

#### 3.2.2. Analyses between the scale scores of four diagnostic groups

**Figure 2:**
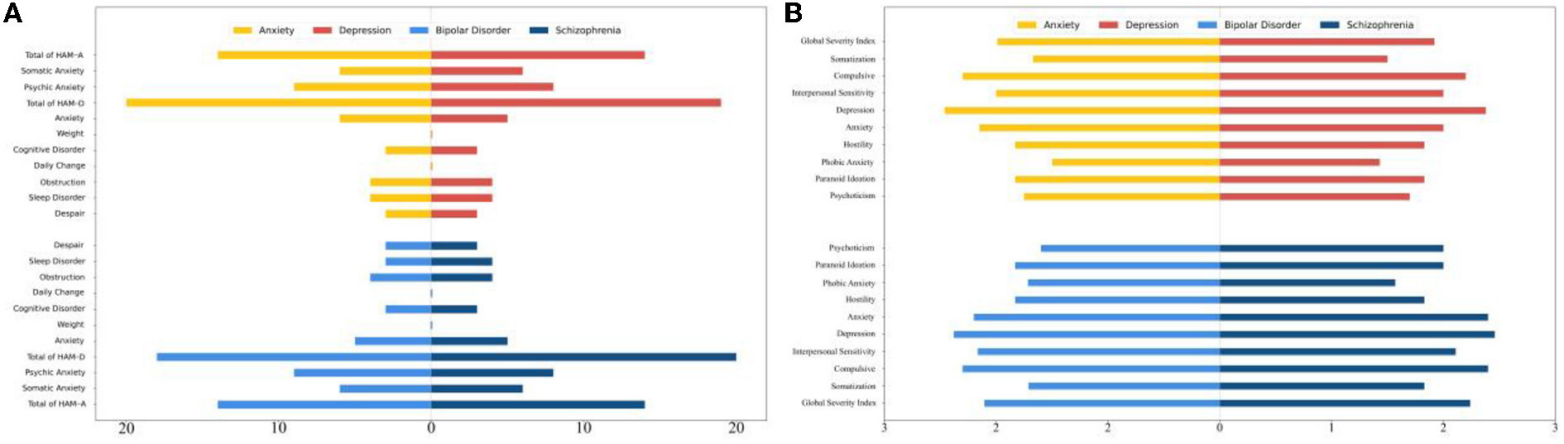
Subscale scores in each diagnostic group. **(A)** Subscale scores and total score of HAM-A and HAM-D in each diagnostic group. **(B)** Subscale scores and total score of SCL-90 in each diagnostic group. HAM-A, Hamilton Anxiety Rating Scale; HAM-D, Hamilton Depression Rating Scale; SCL-90, Symptom Checklist-90.

In each scale, participants were grouped by their diagnoses as group A2 with “anxiety disorder,” group B2 having “depressive disorder,” group C2 with “bipolar disorder,” and group D2 having “schizophrenia.” Bars in Figure 2 showed scores in each diagnostic group. Figure 2A showed subscale scores and total score of HAM-A and HAM-D, and Figure 2B showed subscale scores and GSI score of SCL-90. The Mann–Whitney U test was performed, and there were no significant differences among the four diagnostic groups in all subscales and total scores of HAM-A, HAM-D, and SCL-90 (Table 4).

**Table 4:** Mann–Whitney U test for diagnostic groups.

	**Anxiety**	**Depression**	**Bipolar disorder**	**Schizophrenia**	***P*-value**
**HAM-A**					
Number of records (*n*, %)	427	735	123	918	
Total	14.0 (10.0–20.0)	14.0 (10.0–20.0)	14.0 (10.0–19.0)	14.0 (10.0–20.0)	0.9043
Somatic anxiety	6.0 (3.0–9.0)	6.0 (3.0–8.0)	6.0 (3.0–8.5.0)	6.0 (3.0–9.0)	0.9275
Psychic anxiety	9.0 (6.0–12.0)	8.0 (6.0–12.0)	9.0 (6.0–11.0)	8.0 (6.0–12.0)	0.8389
**HAM-D**					
Number of records (*n*, %)	416	719	122	900	
Total	20.0 (14.0–27.0)	19.0 (13.0–27.0)	18.0 (14.0–27.0)	20.0 (12.0–27.0)	0.4145
Anxiety	6.0 (4.0–8.0)	5.0 (3.0–7.0)	5.0 (3.0–7.0)	5.0 (3.0–7.0)	0.3716
Weight	0 (0–1.0)	0 (0–1.0)	0 (0–1.0)	0 (0–1.0)	0.1250
Cognitive disorder	3.0 (2.0–5.0)	3.0 (2.0–5.0)	3.0 (2.0–5.0)	3.0 (1.0–5.0)	0.6365
Daily change	0 (0–1.0)	0 (0–1.0)	0 (0–1.0)	0 (0–1.0)	0.1953
Obstruction	4.0 (2.0–5.0)	4.0 (2.0–5.0)	4.0 (2.0–5.0)	4.0 (2.0–5.0)	0.6428
Sleep disorder	4.0 (2.0–5.0)	4.0 (2.0–5.0)	3.0 (2.0–6.0)	4.0 (2.0–5.0)	0.4489
Despair	3.0 (1.0–4.0)	3.0 (1.0–4.0)	3.0 (1.0–4.0)	3.0 (1.0–4.0)	0.8047
**SCL-90**					
Number of records (*n*, %)	100	161	30	163	
Global Severity Index	2.0 (1.4–2.9)	1.9 (1.5–2.8)	2.1 (1.6–2.8)	2.2 (1.5–3.0)	0.6105
Somatization	1.7 (1.3–2.5)	1.5 (1.2–2.3)	1.7 (1.3–2.4)	1.8 (1.3–2.6)	0.2661
Compulsive	2.3 (1.4–3.3)	2.2 (1.5–3.0)	2.3 (1.7–3.0)	2.4 (1.6–3.3)	0.5772
Interpersonal sensitivity	2.0 (1.3–3.0)	2.0 (1.4–2.8)	2.2 (1.7–3.7)	2.1 (1.4–3.3)	0.3532
Depression	2.5 (1.4–3.5)	2.4 (1.6–3.5)	2.4 (1.6–3.4)	2.5 (1.6–3.7)	0.7834
Anxiety	2.2 (1.5–3.3)	2.0 (1.4–3.1)	2.2 (1.5–2.9)	2.4 (1.5–3.3)	0.5086
Hostility	1.8 (1.3–3.0)	1.8 (1.3–2.8)	1.8 (1.5–2.9)	1.8 (1.3–2.8)	0.9943
Phobic anxiety	1.5 (1.1–2.6)	1.4 (1.1–2.3)	1.7 (1.1–2.6)	1.6 (1.1–2.4)	0.6389
Paranoid ideation	1.8 (1.2–2.8)	1.8 (1.2–2.5)	1.8 (1.2–3.0)	2.0 (1.3–3.0)	0.3562
Psychoticism	1.8 (1.1–2.8)	1.7 (1.2–2.4)	1.6 (1.3–2.5)	2.0 (1.3–2.7)	0.3327
Additional item-sleep and diet	2.3 (1.6–3.1)	2.1 (1.6–3.0)	2.4 (1.7–2.9)	2.4 (1.7–3.1)	0.4850

HAM-A, Hamilton Anxiety Rating Scale; HAM-D, Hamilton Depression Rating Scale; SCL-90, Symptom Checklist-90.

Data are presented as median and interquartile range (IQR).

Statistical analysis: Mann–Whitney U test.

Given that there were significant differences in most of the subscale scores between age and gender groups, under the same diagnostic grouping method, age (as a continuous variable) and sex (divided into “male” and “female”) were regarded as covariates. The scale scores were dependent variables, whereas the disease groups were independent variables in all the covariance models. Considering the data distribution was not normal, non-parametric test (Kendall's tau and Spearman's r) can provide a better protection against type I errors than Pearson's r. An early study showed that, regarding psychiatric scales, Kendall's tau could maintain more adequate prevention against type I errors and provide more accurate results than Spearman's r (21), thus we conducted Kendall's tau coefficient analyses and found that there was no statistical significance in diseases in the total scale scores and nearly all subscale scores except weight (Kendall = −0.0366, *P* = 0.0109) and daily change (Kendall = −0.0344, *P* = 0.0167) scores of HAM-D (Table 5).

**Table 5:** Kendall's tau coefficient analyses of diagnostic groups.

	**Kendall**	***P*-value**
**HAM-A**		
Total	−0.00879	0.5367
Somatic anxiety	−0.00087	0.9512
Psychic anxiety	−0.01115	0.4331
**HAM-D**		
Total	−0.01896	0.1870
Anxiety	−0.01115	0.4377
Weight	−0.03659	0.0109^*^
Cognitive disorder	−0.00835	0.5611
Daily change	−0.03438	0.0167^*^
Obstruction	−0.0168	0.2425
Sleep disorder	−0.02131	0.1382
Despair	−0.01643	0.2529
**SCL-90**		
Global severity index	0.040213	0.2014
Somatization	0.044032	0.1619
Compulsive	0.043949	0.1627
Interpersonal sensitivity	0.04345	0.1675
Depression	0.037965	0.2278
Anxiety	0.038235	0.2245
Hostility	0.009934	0.7523
Phobic anxiety	0.028227	0.3699
Paranoid ideation	0.03191	0.3107
Psychoticism	0.044181	0.1605
Additional item-sleep and diet	0.029370	0.3508

HAM-A, Hamilton Anxiety Rating Scale; HAM-D, Hamilton Depression Rating Scale; SCL-90, Symptom Checklist-90.

Statistical analysis: Kendall's tau coefficient analyses; ^*^P < 0.05.

#### 3.2.3. Machine learning result

The detailed performance of the models is presented in Table 6. We calculated multiple accuracy indicators, including accuracy score, F-measure (F1), precision score (Prec.), and the area under the receiver operating characteristic curve (AUC). For these indicators, the micro- and macro-averages were calculated. The micro-average does not distinguish among categories and calculates the overall level of accuracy, while the macro-average calculates the indicators of each category separately and weighs the average.

**Table 6:** Detailed performance of models.

**Models**	**Scales**	**Accuracy**	**F1_macro_**	**F1_micro_**	**AUC_macro_**	**AUC_micro_**	**Prec._macro_**
	**HAM-A**						
**SAINT**		0.41	**0.21**	0.40	**0.53**	0.70	**0.30**
FCNN		**0.42**	0.15	**0.42**	0.52	**0.71**	0.10
XGBoost		0.41	0.21	0.41	0.51	0.71	0.19
LR		0.41	0.15	0.40	0.51	0.70	0.14
	**HAM-D**						
SAINT		**0.42**	0.15	**0.42**	0.51	0.70	0.10
FCNN		0.42	0.15	0.42	0.50	0.70	0.10
XGBoost		0.38	**0.20**	0.38	0.51	0.68	**0.21**
**LR**		0.42	0.18	0.42	**0.53**	**0.71**	0.20
	**SCL-90**						
**SAINT**		**0.37**	0.23	**0.37**	**0.56**	**0.67**	**0.25**
FCNN		0.35	0.13	0.35	0.48	0.66	0.09
XGBoost		0.35	**0.26**	0.35	0.47	0.61	0.26
LR		0.33	0.22	0.35	0.52	0.65	0.24

SAINT, Self-attention and Intersample attention transformer; FCNN, Fully connected neural network; LR, Logistic regression; HAM-A, Hamilton anxiety rating scale; HAM-D, Hamilton depression rating scale; SCL-90, Symptom checklist-90.

The bold values indicate the maximum value in each column of each scale.

**Figure 3:**
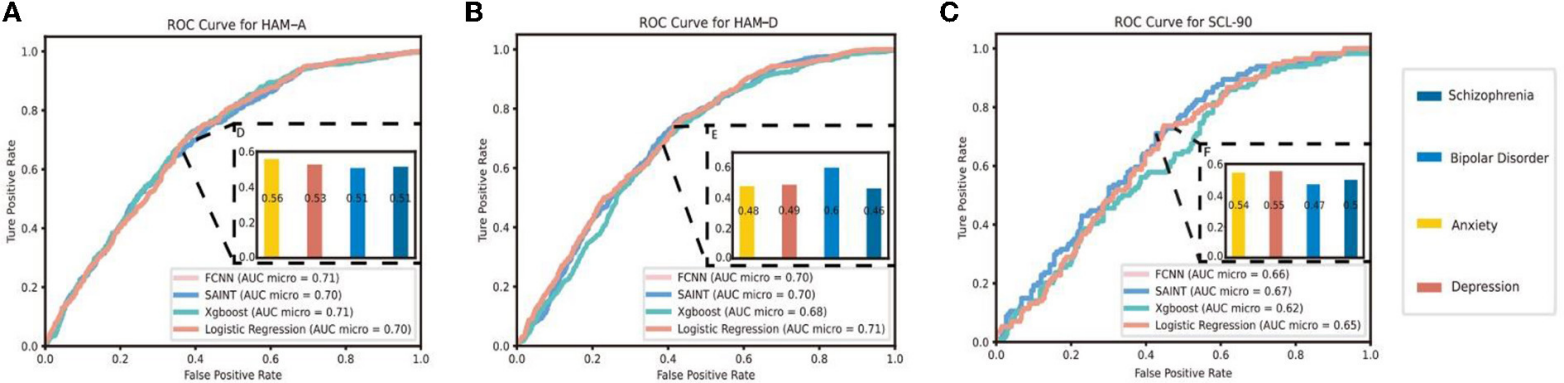
**(A)** AUC and ROC Curve in each diagnostic group for HAM-A. **(B)** AUC and ROC Curve in each diagnostic group for HAM-D. **(C)** AUC and ROC Curve in each diagnostic group for SCL-90. HAM-A, Hamilton anxiety rating scale; HAM-D, Hamilton depression rating scale; SCL-90, Symptom checklist-90; AUC, Area under the receiver operating characteristic curve; ROC, Receiver operating characteristic curve; SAINT, Self-attention and Intersample attention transformer; FCNN, Fully connected neural network.

The model with the highest AUC_macro_ was considered optimal for each scale. The receiver operating characteristic curve (ROC) and AUC of each category of the best model are displayed in Figures 3A–C. We found that these models perform nearly identically on each scale with SAINT slightly better on HAM-A and SCL-90; and LR was the best model trained on HAM-D. We also found that, for HAM-A, anxiety had a small degree of differentiation with an AUC of 0.56 while other diseases had an AUC close to 0.50. As for HAM-D, bipolar disorder was slightly distinguishable with an AUC of 0.60 and the AUC of other diseases was lower than 0.50. Regarding SCL-90, all diseases had similar AUC; among them, bipolar disorder had the lowest (AUC: 0.54), schizophrenia had the highest (AUC: 0.57), while anxiety and depression both had an AUC of approximately 0.56.

## 4. Discussion

Our statistics contained 4,814 records, over a duration of one and a half years, obtained from the Pudong Mental Health Center, which is a mental health specialist hospital located in Shanghai and has professional medical teams. Thus, our data is reliable and represents the practical use of these questionnaires in a real Chinese psychiatrist outpatient clinic environment.

Self-reported SCL-90, clinician-related HAM-A, and HAM-D are all widely used mental questionnaires in China, which are reported to have great potency in screening mental health, diagnosing, and estimating mental illness (22–24). In China, previous studies on SCL-90 focused primarily on students, doctors, nurses, police, and migrant workers, among others (24–27). All three scales were mostly used as tools to evaluate the severity of certain symptoms in a specific patient or in the normal adult group. Although they cannot directly diagnose mental disorders (28), as widely used testing tools in clinics, its value in enabling doctors make better and quicker diagnoses needs to be further explored.

Unlike previous studies, we conducted wide and comprehensive analyses on the use of these three scales in outpatient clinics, mainly focusing on whether there were significant differences in the item scores between different mental illnesses. Our aim was to evaluate whether these questionnaires could provide valuable score differences to help doctors discriminate between different mental illnesses.

As mentioned in our results, females generally had higher subscale scores than males. Compared to males, females tended to have higher levels of anxiety, sleep disorders, and somatization symptoms. In addition, people in early adulthood showed more severe symptoms than those in other age groups. These results are consistent with those of the report from the World Health Organization (WHO), which indicates that globally, young people and women are more easily affected by economic and social events, especially considering the recent COVID-19 pandemic. The results of a previous study on psychological symptoms in Chinese citizens (29) support this view. These findings revealed that when doctors use the scales as auxiliary diagnostic tools, they should consider the differences between different age and gender groups.

We performed the Mann–Whitney U test between the item scores of SCL-90, HAM-A, and HAM-D in anxiety disorder, depressive disorder, bipolar disorder, and schizophrenia, and found no significant differences. Considering the significant differences of the subscales scores between age and gender groups, we adjusted age (as continuous variables) and gender (divided into “male” and “female”) factors as covariates and conducted Kendall's tau coefficient of partial correlation, but the positive results were inadequate, except the weight and daily change subscales of HAM-D, which demonstrated that the use of these scales as auxiliary tools for facilitating doctors to differentiate different mental illnesses and make accurate diagnoses may be limited.

While we confirmed that the univariate subscale scores have no statistical significance among anxiety disorder, depression disorder, bipolar disorder, and schizophrenia, there are no features or patterns that can be extracted from the combination of item scores of SCL-90, HAM-A, and HAM-D that can be used for differential diagnosis. Therefore, we conducted a trial using ML models to evaluate the diagnostic effects of these scales.

As the AUC_macro_ of all scales was approximately 0.5, we found that there was no predictive value in all the scale scores. However, the AUC_micro_ values were relatively higher, and most of them were close to 0.7, indicating a small degree of classification accuracy. The classification abilities of these scales differed according to disease category. In HAM-A, anxiety can be identified with an AUC of 0.56, whereas bipolar disorder can be recognized using HAM-D with an AUC of 0.60. All the diseases could be slightly distinguished using SCL-90.

Symptom Checklist-90 (SCL-90) is a comprehensive scale that establishes evaluation indicators for many psychiatric symptoms included in numerous diseases. As mentioned in our results, there were no statistically significant differences between different disease diagnoses in the subscale scores and mean score of patients, which indicated weak diagnostic effect. A study in China, which researched the norm and application of SCL-90 in the past decades, found that the subscales of SCL-90 could not distinguish mental diseases adequately (25). Another study which investigated the application of SCL-90 in 7489 Chinese also induced that the detective specificity of SCL-90 has reduced (30). These researches further supported the result that SCL-90 could only distinguish the diseases included in our study with low AUC.

Depression and anxiety disorders had high rates of comorbidity in many other mental diseases and had significant correlations (31–33), which indicated the reasonability that HAM-D and HAM-A poorly performed in distinguishing the four disorders in our study. A research of 1,741 patients with Major Depressive Disorder (MDD) found that the “insight” and “genital symptoms” items in HAM-D had poor discrimination, and some scores were even lower when patients had higher depressive severity, thus HAMD-17 was not recommended to assess the severity of depressive patients in outpatient clinics (23). Another study of patients having bipolar depression, bipolar depression with mixed features, or MDD showed that HAM-D17 failed to be unidimensional to distinguish MDD from the others (34). A study conducted in 203 patients with MDD indicated that, as an item of HAM-A could assess depression and some items of HAM-D included the evaluation of anxiety, HAM-D had great correlation with HAM-A (35). Thus, although HAM-D and HAM-A could prove to be sensitive in treatment (36) and distinguishing normal people from patients with mental diseases (37, 38), they had low discrimination in different mental disorders.

The classification ability of ML models has surpassed that of humans in many domains (39–41). Although our experimental trial is not very strict, it can show that the scale scores are not a very strong feature of differential diagnosis among these four mental illnesses. In conclusion, although these widely used mental questionnaires have good reliability and validity according to many classical studies, the degree of differentiation of these scale scores between different diseases is not obvious, which highlights the limit of their practical use by doctors in identifying different mental diseases in Chinese outpatient psychiatric services.

## 5. Limitations

Our study had several limitations. First, we did not conduct a reliability and validity analysis in our study group, which will be conducted in future research. Secondly, as there was a lack of authoritative studies on Chinese population with SCL-90, HAM-A, and HAM-D, which also used quartiles, we could not compare the subscale scores of our study with those of other studies and populations.

## 6. Conclusion

In this study, based on analyzing 4,814 records of commonly used mental questionnaires from the database of the Pudong Mental Health Center, Shanghai, China, we evaluated whether mental questionnaires could provide valuable score differences to help doctors discriminate between different mental illnesses in a realistic hospital environment. We found that there were no significant differences among depressive disorder, anxiety disorder, bipolar disorder and schizophrenia in all subscales and total scores of SCL-90, HAM-D, and HAM-A by using Kendall's tau coefficient analyses. According to machine learning result, the AUC of these four disorders were generally at around 0.50; bipolar disorder had the highest AUC of 0.60 in HAM-D. This paper is the first to combine traditional statistical approach and novel machine learning method to conduct comprehensive analyses on the use of mental scales in Chinese outpatient clinics, and we emphasized the limit of their practical use in identifying different mental disorders.

## Data availability statement

The raw data supporting the conclusions of this article will be made available by the authors, without undue reservation.

## Ethics statement

The study was approved by the Ethics Committee of the Institutional Review Board at Pudong Mental Health Center. Written informed consent for participation was not required for this study in accordance with the national legislation and the institutional requirements. Written informed consent was obtained from the individual(s) for the publication of any potentially identifiable images or data included in this article.

## Author contributions

JW and EZ: experimental design, data acquisition, data analysis, and manuscript writing. PA: manuscript editing. JL: data analysis. ZC: data analysis and picture processing. FW: data acquisition. FC and ZA: experimental design and manuscript revision. All authors contributed to the article and approved the submitted version.
